# VGAE-MCTS: A New Molecular Generative Model Combining the Variational Graph Auto-Encoder and Monte Carlo Tree Search

**DOI:** 10.1021/acs.jcim.3c01220

**Published:** 2023-11-22

**Authors:** Hiroaki Iwata, Taichi Nakai, Takuto Koyama, Shigeyuki Matsumoto, Ryosuke Kojima, Yasushi Okuno

**Affiliations:** † Graduate School of Medicine, Kyoto University, 53 Shogoin-kawaharacho, Sakyo-ku, Kyoto-shi, Kyoto 606-8507, Japan; ‡ HPC- and AI-driven Drug Development Platform Division, RIKEN Center for Computational Science, Kobe-shi, Hyogo 650-0047, Japan

## Abstract

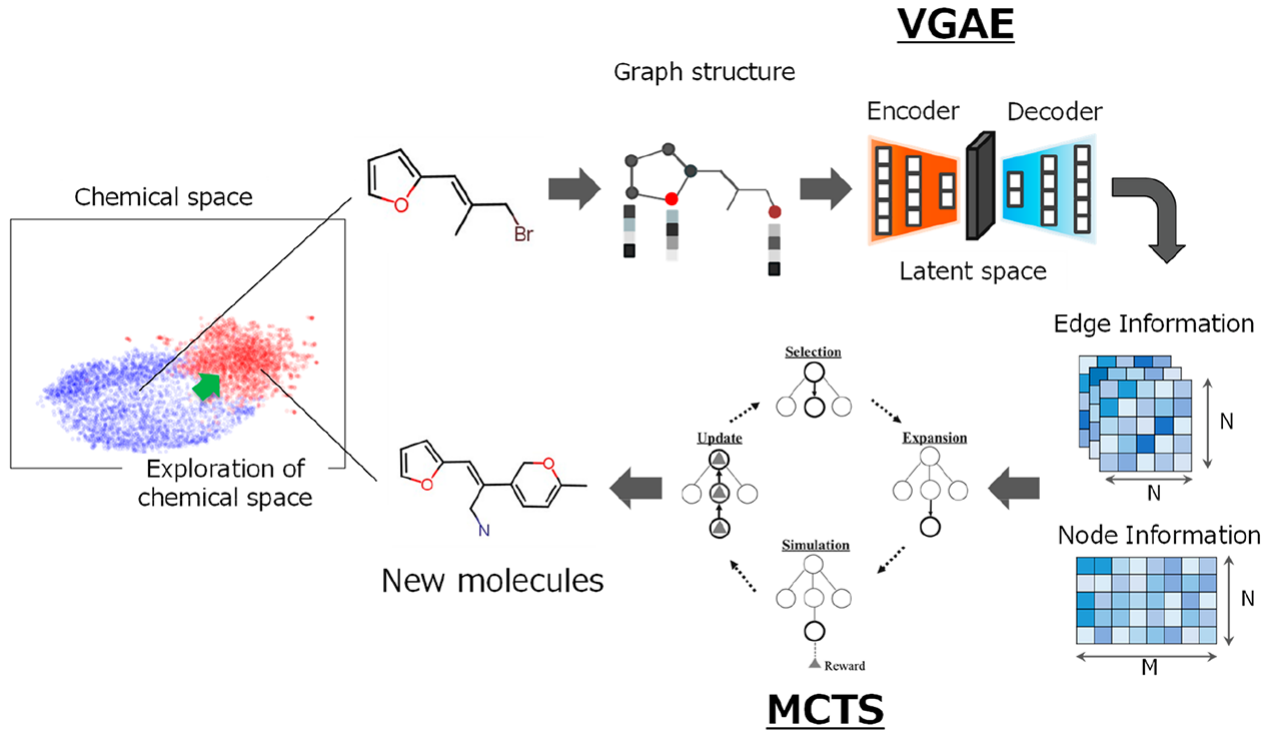

Molecular generation is crucial for advancing drug discovery, materials science, and chemical exploration. It expedites the search for new drug candidates, facilitates tailored material creation, and enhances our understanding of molecular diversity. By employing artificial intelligence techniques such as molecular generative models based on molecular graphs, researchers have tackled the challenge of identifying efficient molecules with desired properties. Here, we propose a new molecular generative model combining a graph-based deep neural network and a reinforcement learning technique. We evaluated the validity, novelty, and optimized physicochemical properties of the generated molecules. Importantly, the model explored uncharted regions of chemical space, allowing for the efficient discovery and design of new molecules. This innovative approach has considerable potential to revolutionize drug discovery, materials science, and chemical research for accelerating scientific innovation. By leveraging advanced techniques and exploring previously unexplored chemical spaces, this study offers promising prospects for the efficient discovery and design of new molecules in the field of drug development.

## Introduction

Molecular generation is crucial for its applications in novel drug discovery, materials science, and the exploration of chemical space. It enables the efficient search and identification of new drug candidates, speeding up the process of drug development.^1,2^ In materials science, it allows for the creation of materials with tailored properties, contributing to advancements in various industries.^3^ Furthermore, molecular generation aids in the systematic exploration of chemical space, uncovering novel compounds with unique properties and expanding our understanding of molecular diversity.^4^ Overall, it has the potential to revolutionize the fields of drug discovery, materials science, and chemical research for accelerating scientific innovation.^3,5^

Although it has been estimated that there are theoretically more than 10^60^ small organic molecule chemical structures,^6^ the number of molecules actually explored in drug discovery is limited to about 10^8^ at most.^7,8^ To efficiently propose new molecules with desirable physicochemical properties from a wide chemical space, an artificial intelligence (AI) technique called molecular generative model has been developed in recent years.^6,9^ As input of the AI model, the chemical structure is represented in three ways: SMILES,^10^ molecular descriptors, and molecular graph.^11,12^ Numerous molecular generation algorithms that utilize the SMILES format as input has been reported.^13−17^ Specifically, owing to the string-based nature of SMILES representation, it aligns well with language models. Consequently, a multitude of approaches employing the Transformer,^18^ which has garnered recent success, has been reported.^13,14^ The approach to molecule generation that utilizes molecular descriptor formats as input is known as fragment-based topological design. Numerous studies have already been conducted, focusing on this approach within various research areas of drug discovery, with a particular emphasis on anticancer drug research^19−23^ and antibacterial drug discovery.^24−28^ In general, molecular graphs are more robust and precise for representing molecular features than SMILES and molecular descriptors because graph representations can capture molecular similarities and perform chemical checks, such as protecting the number of valence electrons, unlike SMILES and molecular descriptors representations.^29,30^ With these advantages, chemical structures represented as molecular graphs have been reported to work well in many cheminformatics studies.^29−33^ In this study, we focused on a graph representation for molecules.

The two main approaches to molecular generative models are deep learning and reinforcement learning.^29,34−36^ Since deep learning-based models, which learn molecular features of known compounds,^29,34^ tend to generate molecules similar to the learned compounds, the ability to generate structurally new compounds is fundamentally limited.^37,38^ In contrast, reinforcement learning-based models, which learn molecular features from scratch without prior learning of known compounds, are superior in generating molecules with structures distinct from known compounds.^35,36^ However, the generated molecules fundamentally lack drug-like properties because the algorithm explores a chemical space distinct from that of the existing compounds. The molecules produced by the two types of molecular generative models would be located far apart from each other in the chemical space, indicating that there is a region beyond the reach of exploration between the two groups of molecules.

**Figure 1:**
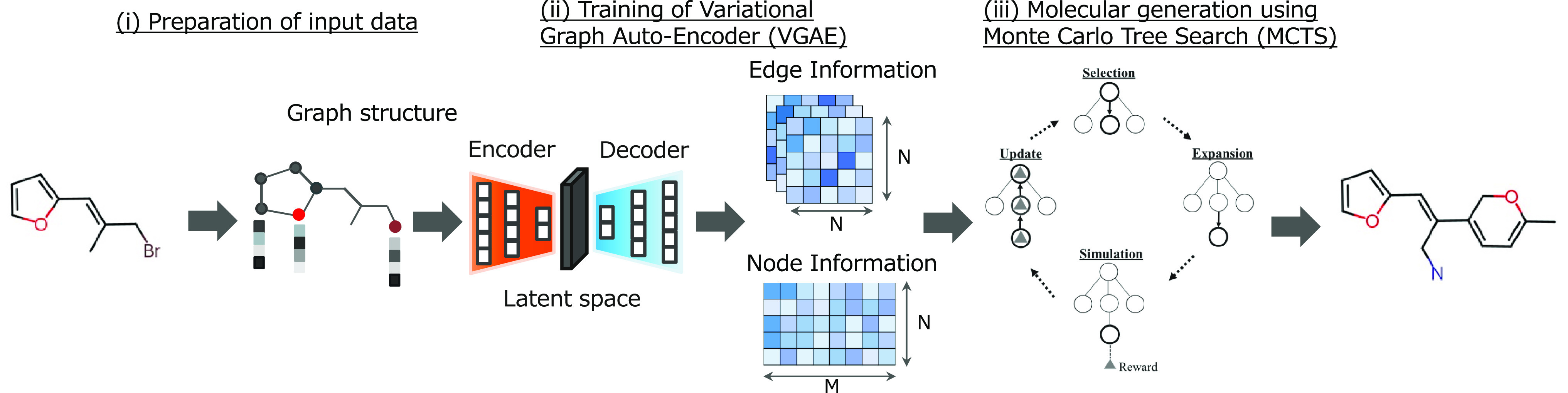
Workflow of our proposed model (VGAE-MCTS). VGAE-MCTS consists of three parts: (i) Converting the molecules of the training data into feature maps (preparation of input data), (ii) Training the distribution of molecules in the training data using the VGAE (training of Variational Graph Auto-Encoder, VGAE), and (iii) Generating molecules by connecting atoms and bonds one by one based on the feature map output from the learned VGAE decoder using MCTS (molecular generation using Monte Carlo Tree Search, MCTS).

In this study, we proposed a new molecular generative model that can explore chemical spaces unreachable by previous molecular generative models and discover new molecules with drug-like properties by combining deep learning and reinforcement learning based on a molecular graph representation (Figure 1). Specifically, the proposed model uses chemical features that learn the physicochemical properties of known compounds using the Variational Graph Auto-Encoder (VGAE)^39^ and generates molecules with desirable properties via reinforced learning with Monte Carlo Tree Search (MCTS).^40^ Evaluation of the generated molecules demonstrated that the validity and novelty of the chemical structures and the optimization of physicochemical properties were equivalent to or better than those from previous models. Furthermore, investigating the chemical structure diversity showed that the generated molecules are distributed in a chemical space that was not well-explored by previous models. The proposed model is expected to be useful for efficiently discovering and designing new molecules in drug development and materials science.

## Materials and Methods

### Data

Two compound data sets were prepared for the training of the VGAE in the proposed model. The first data set comprised compounds obtained from ChEMBL^41^ for the evaluation of basic molecular generating capability (validity, uniqueness, novelty, Kullback–Leibler (KL) divergence, and Fréchet ChemNet Distance (FCD)) using GuacaMol’s Distribution-Learning Benchmarks.^42^ The total number of compounds obtained from ChEMBL was 1,352,672, which were divided into 1,273,104 for training and 79,568 for validation. As the second data set, compounds were obtained from ZINC,^43^ where drug-like compounds are registered, to evaluate the molecular generation capability to optimize the physicochemical properties of molecular generation. The number of compounds obtained from ZINC was 249,456, which were divided into 199,565 for training and 49,891 for validation.

### Proposed Models

#### Preparation of Input Data

The molecules of the training data set are represented in a molecular graph, where atoms are represented by nodes and bonds by edges with features. In the conversion of the molecules to graph representation with feature vectors, node features and edge features were computed using RDKit.^44^ Details of node and edge features are shown in Supplementary Tables 2 and 3, respectively. We call a matrix concatenated with the all node features a feature map of nodes and matrices concatenated with the all edge features a feature map of the edges.

#### Training of the Variational Graph Auto-Encoder (VGAE)

The VGAE was used to generate feature maps for use in MCTS. The VGAE consists of an encoder (Supplementary Figure 1 left) and a decoder (Supplementary Figure 1 right).^45^ The loss function of the VGAE is designed using the evidence lower bound in a variational inference framework. More concretely, this loss function consists of a regularization term calculated by the KL divergence between the normal distribution with mean 0 and variance 1 and the distribution of the encoder’s output and the sum of the reconstruction error calculated based on the input data and the data output by the decoder. For the training of the VGAE in our proposed model, a latent space of 64 dimensions, a learning rate of 0.001, and a batch size of 64 were used.

#### Molecular Generation Using Monte Carlo Tree Search (MCTS)

Molecules are generated by connecting atoms and bonds one by one in MCTS based on the feature map output by the learned VGAE. Latent variables are randomly selected from the latent space of the learned VGAE. The selected latent variables are passed through a decoder to output a feature map of edges, which represents the probability of existence of atom–atom edges, and a feature map of nodes, which represents the features of atoms.

MCTS generates molecules using the feature maps output from the trained VGAE. In MCTS, the following, 1) Selection, 2) Expansion, 3) Simulation, and 4) Update, are considered one search and repeated for the number of times specified by the user. When the search is completed for the number of times specified by the user on one feature map, the molecule with the best physicochemical property value at each depth of the MCTS is output for numerators below the user-specified depth (*minimum_depth*). Then, the search moves on to the next feature map.

1) Selection: Select one node that has the smallest value in the following equation.
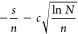
1where *s* is the score of the node, *n* is the number of times the node has been visited, *c* is the search coefficient (*c* = 1.5 in our case), and *N* is the number of times the parent node has been visited. Eq 1 corresponds to Equation of the Upper Confidence Bound 1,^46^ which is well-known in reinforcement learning. At this time, the depth is increased by one with the selected node.
2) Expansion: Bonding and atom addition are performed based on the candidate edges for the selected node (molecule).
3) Simulation: Roll out the molecules to which bonds and atoms were added in the Expansion part.
4) Update: The node is updated with a reward based on the physical properties of the molecule after the rollout.

The threshold and the number of iterations for candidate edge extraction for this model were set to 0.10 and 8,000. For minimum_depth, we set it to 21 for the GuacaMol benchmark measurement, 17 for the Quantitative Estimate of Druglikeness (QED) optimization, and 6 for the penalized log *P* optimization.

In our model, the “aromatic force cycle mode” was introduced to facilitate the formation of aromatic rings, which are important for drug discovery. The “aromatic force cycle mode” has the following procedures: 1) to 3).

1) The feature map output by the VGAE is used to determine if it is possible to form aromatic rings of the specified size. In this study, aromatic ring sizes were set to 5- and 6-membered rings.
2) If it is determined that aromatic rings can be formed, aromatic rings are generated at the beginning of MCTS.
3) Increase the reward value of nodes for molecules with aromatic rings by 0.5 to make them more likely to be selected than nodes for molecules without aromatic rings during MCTS Selection.

In addition, to generate realistic molecules, the proposed model introduced two filters, a steric strain filter^30^ and a filter, to make it difficult to create ring structures larger than 7-membered rings. If a node was trapped by at least one of these two filters, our model made it less likely to be selected as a node to be searched by increasing the MCTS reward value by a factor of 10.

### Performance Evaluation of Molecular Generation

#### GuacaMol Benchmarks

Distribution-Learning Benchmarks within the GuacaMol framework were used to evaluate five indicators: validity, uniqueness, novelty, KL divergence, and FCD. The scores all range from 0 to 1; the closer the value is to 1, the better the score.

#### Optimization of Physicochemical Properties

The QED^47^ and penalized log *P* were set as the physicochemical properties to be optimized. QED is a quantitative measure of drug-likeness^47^ and ranges from 0 to 1, with values closer to 1 indicating that the molecule is more drug-like. When QED was optimized, the 1-QED score was used as the reward function for MCTS.

Penalized log *P* is a measure that combines three physicochemical properties: lipophilicity (log *P*), ease of synthesis (SA score), and penalty for large rings (RingPenalty). The formula used in the penalized log *P* optimization is defined below^29,48^
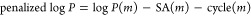
2
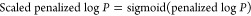
3where *m* denotes the numerator. Using eq 3, penalized log *P* was converted to a range of 0 to 1 to be the score for penalized log *P* optimization; molecules closer to 1 indicate better molecules. When penalized log *P* was optimized, the 1-sigmoid(penalized log *P*) score was used as the reward function for MCTS.

In the evaluation of the optimization of the physicochemical properties, 3000 molecules were randomly selected from the ZINC data set, and 3,000 molecules were selected in the order in which they were generated by each molecule generation model. The distribution of the physicochemical properties for the generated molecules was then calculated and evaluated.

#### Statistical Analysis

The Mann–Whitney U test^49^ was used to test for differences in the distribution of physicochemical property values between models for molecules generated by optimizing QED and penalized log *P*. In addition, the significance probability p-values were corrected by Bonferroni’s correction.^50^ Five hundred randomly selected molecules from the 3,000 molecules generated for each model were used for the test.

#### Visualizing Chemical Space

Molecules from the ZINC data set were used as training data, and molecules generated by optimizing QED with each model were mapped to a chemical space. Molecules were mapped in a two-dimensional space by calculating 2048 dimensional ECFP descriptors^51^ with a diameter of 4 in RDKit and then performing dimensionality reduction using UMAP^52^ in ChemPlot.^53^

## Results

### Proposed Model for Molecular Generation

We developed a new molecular generative model that combines a deep learning model, VGAE, and a reinforcement learning model, MCTS. Our developed model (called VGAE-MCTS) consists of three parts: preparation of input data, training of the VGAE, and molecular generation using MCTS (Figure 1). Details of each part are described in the Materials and Methods section.

### Performance Evaluation

The basic performance of molecular generation using VGAE-MCTS, namely validity, uniqueness, novelty, Kullback–Leibler divergence (KL divergence), and FCD, was evaluated with Distribution-Learning Benchmarks in the GuacaMol framework.^42^ We compared the performance of VGAE-MCTS with the previous models, Graph MCTS^54^ and VGAE^55^ (Table 1).

**Table 1:** Benchmarking Results Using GuacaMol Distribution-Learning Benchmarks

	GraphMCTS^a^	VGAE^a^	VGAE-MCTS
Validity	1.000	0.830	1.000
Uniqueness	1.000	0.944	1.000
Novelty	0.994	1.000	1.000
KL divergence	0.522	0.554	0.659
FCD	0.015	0.016	0.009

a Values of GraphMCTS and VGAE are taken from Table 4 in Mahmood et al.^56^

The molecules generated by VGAE-MCTS showed scores of 1.000 for validity, uniqueness, and novelty. In other words, all molecules generated were valence electron counts protected, not duplicated, and novel molecules that were not present in the training data set. The results for these three types of scores were comparable to or better than those of the previous models. The KL divergence score for VGAE-MCTS was 0.659. This is the highest result compared to that obtained using the previous models Graph MCTS and VGAE. The details of the KL divergence scores for VGAE-MCTS are shown in Supplementary Table 1. The FCD score for the VGAE-MCTS was 0.009. FCD compares the similarity of the distribution of predicted bioactivity values between the generated molecules and compounds from the ChEMBL database. Similar to the previous models, VGAE-MCTS also had a low FCD score.

### Optimizing Physicochemical Properties

The ability of the VGAE-MCTS to generate molecules was evaluated when the physicochemical properties were optimized. The physicochemical properties to be optimized were Quantitative Estimate of Drug-likeness (QED)^47^ and penalized log *P*. QED is a method for quantitatively assessing the drug-likeness of compounds. It is widely used in the fields of medicinal chemistry and drug design. Penalized log *P* is an index that combines three physicochemical properties: liposolubility, synthetic accessibility score, and penalty for large rings, and is an evaluation of the ability to optimize multiproperties. Both indices are commonly used physicochemical properties of drug discovery in the evaluation of molecular generation models.^30,35^ These indices range from 0 to 1, with molecules closer to 1 indicating that they are better molecules for drug discovery. We compared the performance of VGAE-MCTS with the prior study models, Junction Tree Variational Autoencoder (JT-VAE)^29^ and Molecule Deep Q-Networks (MolDQN).^35^

### QED Optimization

**Figure 2:**
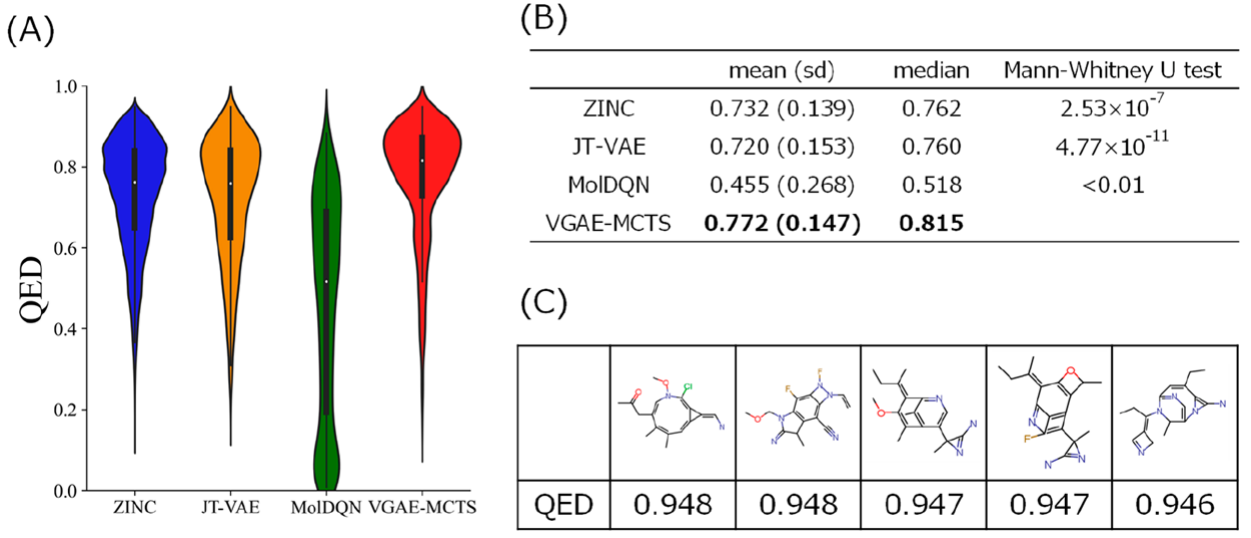
Results of QED-optimized generated molecules. (A) The vertical axis shows the QED value from 0 to 1, and the horizontal axis shows the molecules of the ZINC data set, previous models, and VGAE-MCTS. White dots represent the mean values, and the bulge represents the density. (B) Mean (standard deviation) and median QED values for each molecular group are shown. (C) Chemical structures of the top five molecules generated by VGAE-MCTS and their QED values are displayed.

Molecules generated by optimizing the QED score using VGAE-MCTS were compared with molecules from the ZINC data set used as training data. In addition, comparisons were also performed with molecules generated by JT-VAE and MolDQN. The distribution of QED scores for each model is shown in Figure 2(A), and the mean, standard deviation, and median statistics are shown in Figure 2(B). Examples of molecules generated by VGAE-MCTS are shown in Figure 2(C) and Supplementary Figure 2. The QED scores of the molecules generated by VGAE-MCTS (mean: 0.772, median: 0.815) were clearly higher than those in the ZINC data set (mean: 0.732, median: 0.762; Mann–Whitney U test: P = 2.53 × 10^–7^). This result suggests that VGAE-MCTS can expand molecules toward better QED, which is a physicochemical property in the MCTS-based search. VGAE-MCTS could also generate higher QED scoring molecules than the previous models JT-VAE (mean: 0.720, median: 0.760) and MolDQN (mean: 0.455, median: 0.518; Mann–Whitney U test: P = 4.77 × 10^–11^, *P* < 0.01).

### Penalized log *P* Optimization

**Figure 3:**
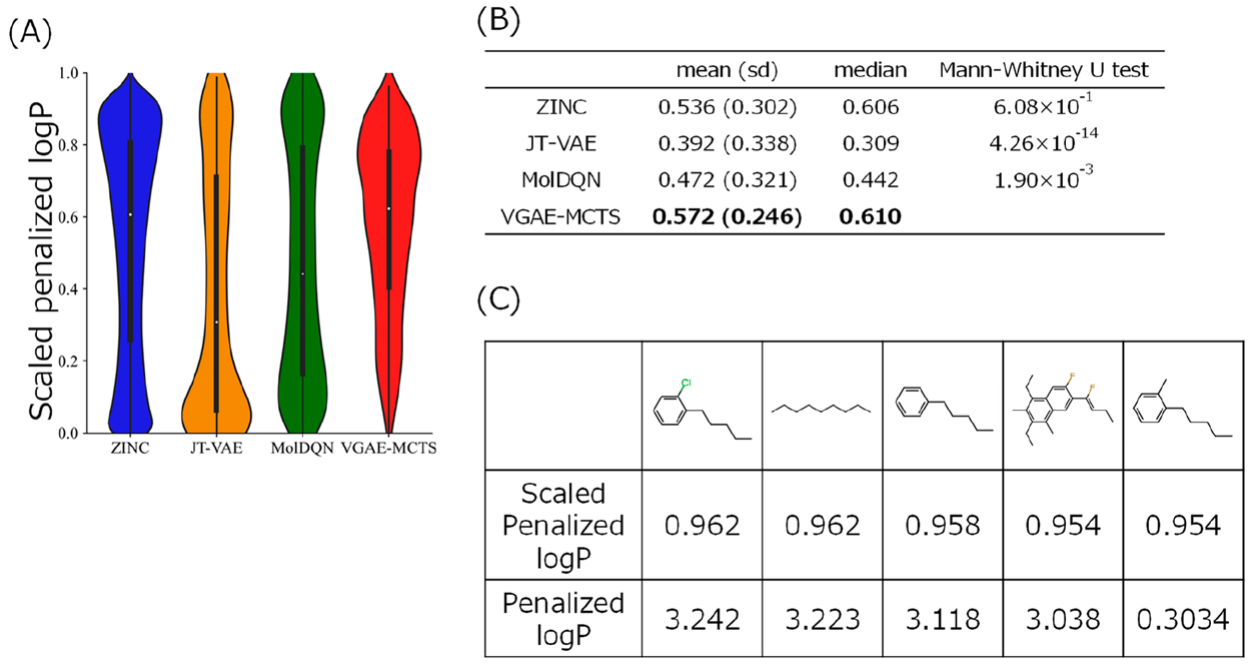
Results of penalized log *P*-optimized generated molecules. (A) The vertical axis shows the scaled penalized log *P* value from 0 to 1, and the horizontal axis shows the molecules of the ZINC data set, previous models, and VGAE-MCTS. The white dots represent the mean scaled penalized log *P* values, and the bulge represents the density. (B) The mean (standard deviation) and median scaled penalized log *P* value for each molecular group are shown. (C) Chemical structures of the top five molecules generated by VGAE-MCTS and their scaled penalized log *P* and penalized log *P* values are displayed.

As with the QED optimization, we compared the molecules generated by optimizing penalized log *P* using VGAE-MCTS with the molecules in the ZINC data set and molecules generated using JT-VAE and MolDQN (Figures3(A), (B), and (C)). The penalized log *P* values of the molecules generated by VGAE-MCTS (mean: 0.536, median: 0.606) were higher than those in the ZINC data set (mean: 0.572, median: 0.610; Mann–Whitney U test: P = 6.08 × 10^–1^). We found that the molecules generated by VGAE-MCTS had a smaller percentage of low penalized log *P* values than those in the ZINC data set and by the previous models. In other words, this suggests that VGAE-MCTS avoids expanding molecules toward the lower penalized log *P* in molecular generation using MCTS. VGAE-MCTS could also generate molecules with higher penalized log *P* values than those obtained using previous models JT-VAE (mean: 0.392, median: 0.309) and MolDQN (mean: 0.472, median: 0.442; Mann–Whitney U test: P = 4.26 × 10^–14^, P = 1.90 × 10^–3^).

### Visualizing the Chemical Space of Generated Molecules

**Figure 4:**
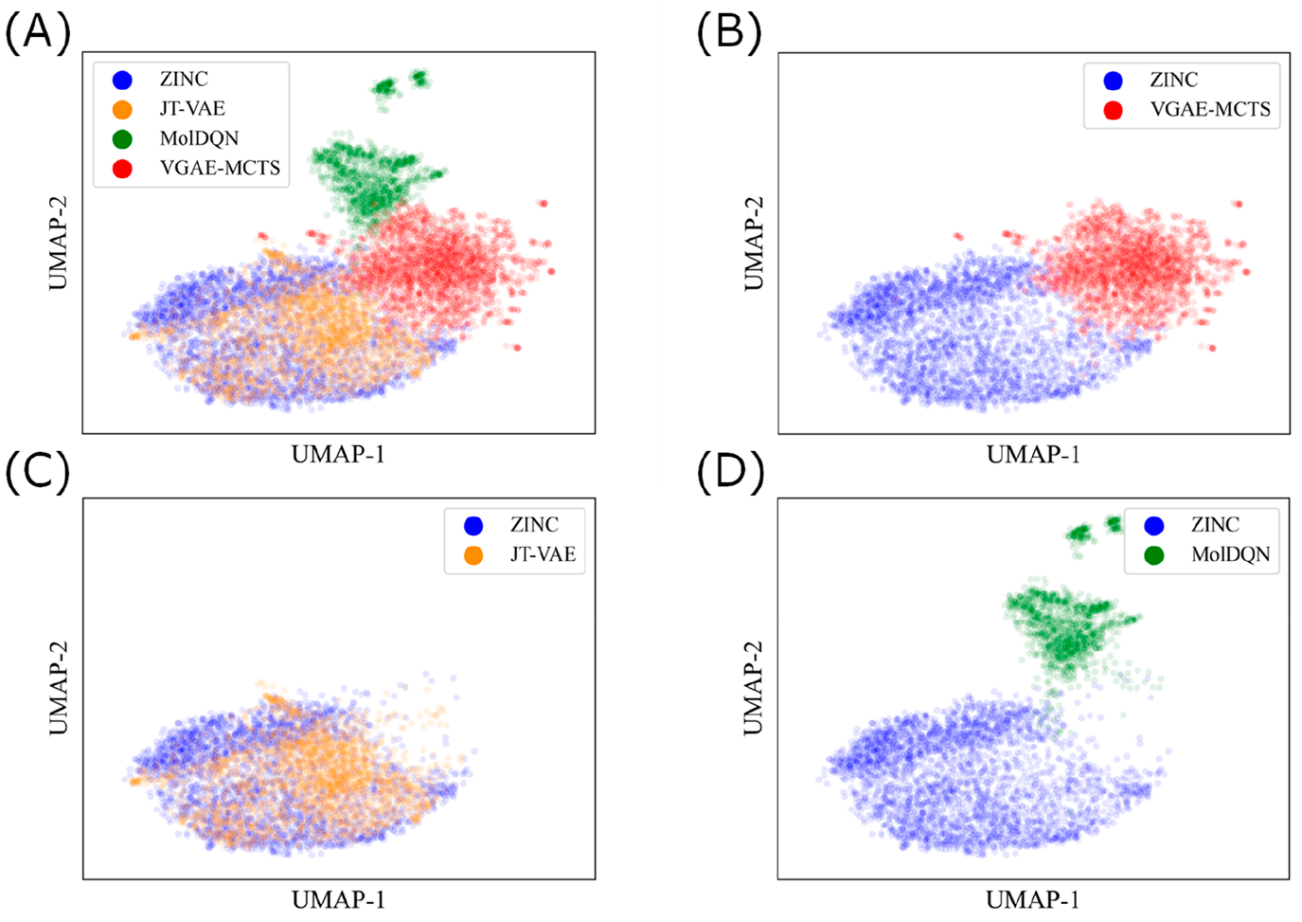
Visualization of the chemical spaces of QED-optimized generated molecules. Molecules generated by optimizing QED are plotted in two dimensions using ECFP. Molecules from the ZINC training data are shown in blue. The molecules generated by JT-VAE are shown in orange, MolDQN in green, and VGAE-MCTS in red. (A) Distribution of molecules in ZINC training data and molecules generated by the three models. (B) Distribution of molecules in ZINC training data and molecules generated by VGAE-MCTS. (C) Distribution of molecules in ZINC training data and molecules generated by JT-VAE. (D) Distribution of molecules in ZINC training data and molecules generated by MolDQN.

We evaluated whether the molecules generated by VGAE-MCTS could expand the chemical space from that of the training molecules. Specifically, molecules generated by each of the QED-optimized models (JT-VAE, MolDQN, and VGAE-MCTS) and molecules from the ZINC data set were mapped onto the chemical space (Figure 4(A)). First, the molecules generated by VGAE-MCTS were plotted in a slightly different chemical space than those in the ZINC data set (Figure 4(B)). In other words, the molecules generated by VGAE-MCTS had a new chemical structure that was slightly different from that of the training molecules. In contrast, the molecules generated by JT-VAE were plotted in almost the same chemical space as the molecules in the ZINC data set (Figure 4(C)). In other words, the molecules generated were very chemically similar to those in the ZINC data set. Molecules generated by MolDQN, which were trained without using the data set, were plotted in a chemical space that was significantly different from those in the ZINC data set (Figure 4(D)).

## Discussion

In this study, our proposed new molecular generative model, VGAE-MCTS, was developed by combining the VGAE, a deep learning model, and MCTS, a reinforcement learning model, to explore chemical spaces that could not be explored by previous models.

First, the basic performance of VGAE-MCTS in generating molecules was evaluated with the distribution learning benchmarks in the GuacaMol framework. The molecules generated by VGAE-MCTS had a validity of 100%. This result is attributed to the fact that the chemical structure is represented as a molecular graph and the MCTS creates molecules by connecting atoms and bonds while protecting the number of valence electrons. The uniqueness and novelty scores of VGAE-MCTS were also higher than those of previous models. These results output a wide variety of molecules because the more atoms that make up a molecule, the more molecules that are candidates for expansion, and the type of atoms selected is stochastic.

Next, molecular generation was performed using VGAE-MCTS to optimize for each of the two types of physicochemical properties values, QED and penalized log *P*, and in both cases, the accuracy was confirmed to be equal to or better than that of previous studies. The molecules generated by QED optimization were more drug-like than those generated by the previous models, indicating that VGAE-MCTS is a valuable model for use in drug discovery. Penalized log *P* is composed of a combination of the three physicochemical properties of log *P*, SA score, and RingPenalty; and multiproperty optimization was relatively successful in VGAE-MCTS. This suggests that VGAE-MCTS can be used to search for molecules considering multiple physicochemical properties. VGAE-MCTS can be expected to be used in practical drug discovery and materials science, where multiple conditions are optimized.

Then, structural and physicochemical analyses were conducted on the molecules generated by optimizing QED. First, a structural interpretation was performed using the molecules generated by each method with the ZINC compounds utilized for training. Similarity scores were calculated using the 2,048-dimensional descriptors of ECFP4 with the molecules generated by each method and 3,000 randomly picked ZINC compounds. The Tanimoto similarity with the most closely related ZINC compound was employed as the similarity score for each generated molecule. The molecules generated by VGAE-MCTS were found to be closer to the training set than those of MolDQN and different from the training set than those of JT-VAE (Supplementary Figure 3(A)). This is consistent with the compound space results in Figure 4. Thus, this outcome suggests the capability of VGAE-MCTS to generate molecules that deviate from the training set, thereby expanding the compound space. Second, a physicochemical analysis was conducted by assessing the molecular weight and the number of aromatic rings in the generated molecules. Of the 3,000 molecules generated by optimizing the QED, we counted how many had a QED greater than 0.9. The outcomes were as follows: 432 molecules for VGAE-MCTS, 195 molecules for JT-VAE, and none for MolDQN. It is noteworthy that MolDQN had its QED optimized, yet it failed to generate any molecules with a QED exceeding 0.9. We compared 3,000 randomly picked ZINC compounds with generated molecules with QED greater than 0.9. The results of the molecular weight comparison are shown in Supplementary Figure 3(B). The molecules generated by VGAE-MCTS and JT-VAE were found to be slightly smaller than those of the ZINC compounds. However, the molecules generated were within the distribution of molecular weights of the ZINC group of compounds, which is an acceptable range. In VGAE-MCTS, the number of iterations was set to 8,000 for the number of MCTS searches in combination with the calculation time. Therefore, it is thought that molecules with slightly smaller molecular weights were generated. Given the availability of additional computation time, it could be possible to increase the number of iterations to generate larger molecules. The results of the log *P* value comparison are shown in Supplementary Figure 2(C). Similar to the molecular weight comparison, the molecules generated by JT-VAE and VGAE-MCTS exhibited log *P* values within the range of the distribution observed in the ZINC compounds. The results regarding the number of aromatic rings present in the generated molecules are shown in Supplementary Figure 3(D). The average number of aromatic rings within the ZINC compounds was 2.75, while the molecules produced by VGAE-MCTS and JT-VAE had average numbers of 2.65 and 2.85, respectively. We considered the results of the three methods to be comparable, in terms of the number of aromatic rings. Third, we presented a sample of molecules generated by VGAE-MCTS in Supplementary Figure 2. For molecules with a QED score greater than 0.9, we further selected those meeting the following criteria: they adhered to Lipinski’s rule of five,^57^ a synthetic accessibility score^58^ of 5 or lower for synthesizability, a steric strain filter^59^ with a cutoff of 0.82 (default) for stability, a log *P* value of 5 or lower, and a ring size of 6 or fewer. Using these criteria, we displayed the selected molecules, sorted by a high QED score. Notably, it was confirmed that molecules relatively close to known pharmaceuticals were also generated.

Finally, we evaluated whether the molecules generated by VGAE-MCTS could expand the chemical space from the training data. As the ZINC data set is registered for drug-like compounds, many molecules have a large QED; approximately 92% of the molecules in the ZINC have a QED ≥ 0.5. Therefore, we can evaluate whether the generated molecules have expanded their chemical space using the chemical space of drug-like molecules in the ZINC as a reference. Figure 4 shows that the molecules generated by VGAE-MCTS were different in structure from those in the ZINC data set. In other words, the molecules generated by VGAE-MCTS showed a chemical spatial spread in the form of derivatives from molecules in the ZINC data set. In contrast, molecules generated by the deep learning-based JT-VAE showed little chemical spatial spread. The molecules generated by the reinforcement learning-based MolDQN were found to be located in a completely different chemical space than those in the ZINC data set; i.e., they were not drug-like molecules. The above confirms that the molecules generated by VGAE-MCTS were located in a part of the chemical space different from those generated by other models. In particular, the molecules generated by VGAE-MCTS were in a part of the chemical space that had not been explored by previous models.

## Conclusion

We developed a new molecular generation model, VGAE-MCTS, which combines VGAE, a deep learning model, and MCTS, a reinforcement learning model, to explore chemical spaces that could not be explored by the models in previous studies. VGAE-MCTS showed comparable or better performance than the existing models in the GuacaMol benchmark. We also showed that the performance of the optimization of the physicochemical properties, QED and penalized log *P*, was comparable to or better than those in previous studies. In addition, to assess the diversity of chemical structures generated, we evaluated the distribution of molecules generated by the VGAE-MCTS and several previous models in chemical space. The results indicate that the molecules generated by VGAE-MCTS are distributed in areas that were not well-explored by the molecules generated by previous models. Based on these results, it is expected that our proposed VGAE-MCTS will be able to produce molecules that may have been out of the scope of exploration so far, which will be useful for drug development and materials science.

## Data Availability

Data and code are provided at the online public link https://github.com/clinfo/VGAE-MCTS.

## Supporting Information Available

Supplementary Table 1. The detail of KL divergence; Supplementary Table 2. Node features of a graph representation; Supplementary Table 3. Edge features of a graph representation; Supplementary Figure 1. Model structures of Encoder and Decoder; Supplementary Figure 2. A hundred molecules generated by VGAE-MCTS with a QED of 0.9 or higher, starting from those with the highest QED scores; Supplementary Figure 3. The results of structural and physicochemical analyses of molecular generative models (PDF)

## Abbreviations

AI: Artificial Intelligence
FCD: Fréchet ChemNet Distance
KL: Kullback–Leibler
MCTS: Monte Carlo Tree Search
QED: Quantitative Estimate of Drug-likeness
VGAE: Variational Graph Auto-Encoder
