# Supplementary material for: VGAE-MCTS: A New Molecular Generative Model Combining the Variational Graph Auto-Encoder and Monte Carlo Tree Search
Source: J Chem Inf Model. 2023 Nov 22;63(23):7392–400. doi: 10.1021/acs.jcim.3c01220 (PMC10716893; doi:10.1021/acs.jcim.3c01220)
Supplement: Supplementary file 1 — ci3c01220_si_001.pdf [file ci3c01220_si_001.pdf]

Supporting Information

VGAE-MCTS: A New Molecular Generative Model Combining Variational Graph Auto-Encoder and Monte Carlo Tree Search

Hiroaki Iwata[1], Taichi Nakai[1], Takuto Koyama[1], Shigeyuki Matsumoto[1], Ryosuke Kojima[1], and Yasushi Okuno[1,2*]*

[1] Graduate School of Medicine, Kyoto University, 53 Shogoin-kawaharacho, Sakyo-ku, Kyoto-shi, Kyoto 606-8507, Japan

[2] HPC- and AI-driven Drug Development Platform Division, RIKEN Center for Computational Science, Kobe-shi, Hyogo 650-0047, Japan

***Corresponding Authors**

Ryosuke Kojima - Email: kojima.ryosuke.8e@kyoto-u.ac.jp

Yasushi Okuno - E-mail: okuno.yasushi.4c@kyoto-u.ac.jp

Supplementary Table 1. The detail of KL divergence

Items	VGAE-MCTS
BertzCT	0.447
LogP	0.903
Molecular weight	0.289
TPSA	0.805
Number of H acceptors	0.958
Number of H donors	0.889
Number of rotatable bonds	0.829
Number of aliphatic rings	0.616
Number of aromatic rings	0.220
Internal similarity	0.637

Supplementary Table 2. Node features of a graph representation

Features	Dimension
Atom type	ChEMBL: 13/ ZINC: 10
Bond order	11
Number of valence electrons	7
Formal charge	1
Number of radical electrons	1
Hybrid orbitals	5
Aromaticity	1
Involvement in ring formation	1
Involvement in 3- to 7-membered ring formation	5
Total number of Hs	5

Supplementary Table 3. Edge features of a graph representation

Features	Dimension
Single bond	1
Double bond	1
Triple bond	1
Aromatic bond	1
Conjugate	1
others	1

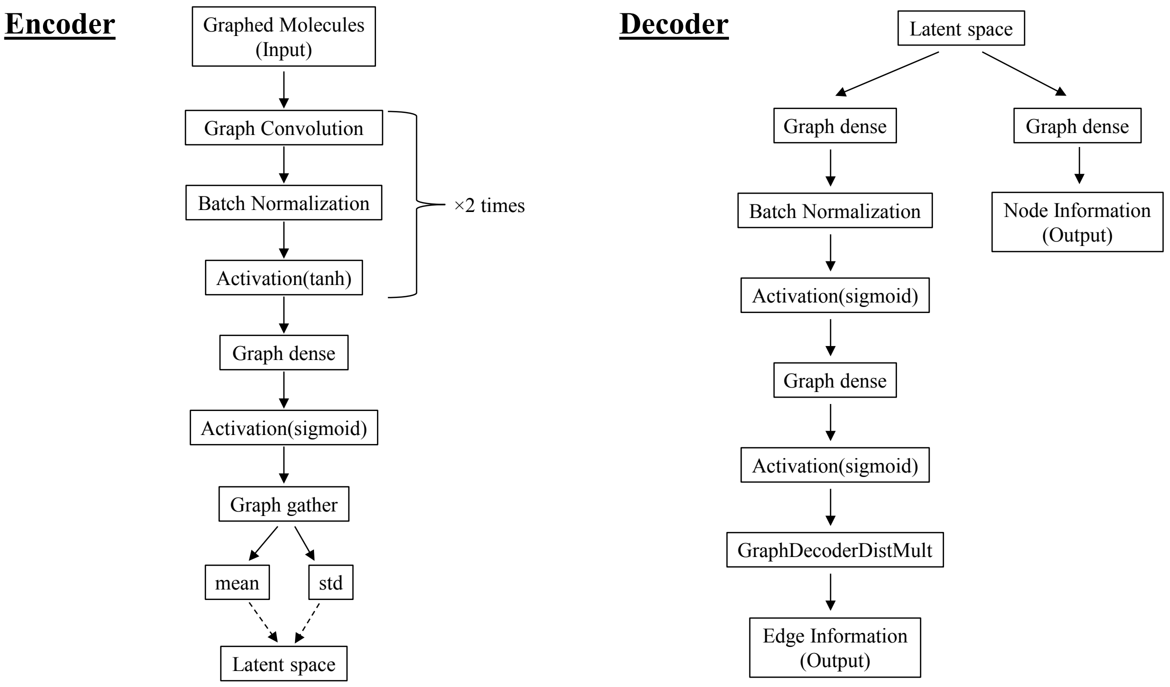

Supplementary Figure 1. Model structures of Encoder and Decoder. Encoder: Considering the entire molecular structure of the GCN model, feature maps are represented by compressing information as low-dimensional vectors and embedding them in the latent space. Decoder: From the distribution in the latent space, the information of the molecules compressed into a low-dimensional vector is recovered in the form of a feature map.

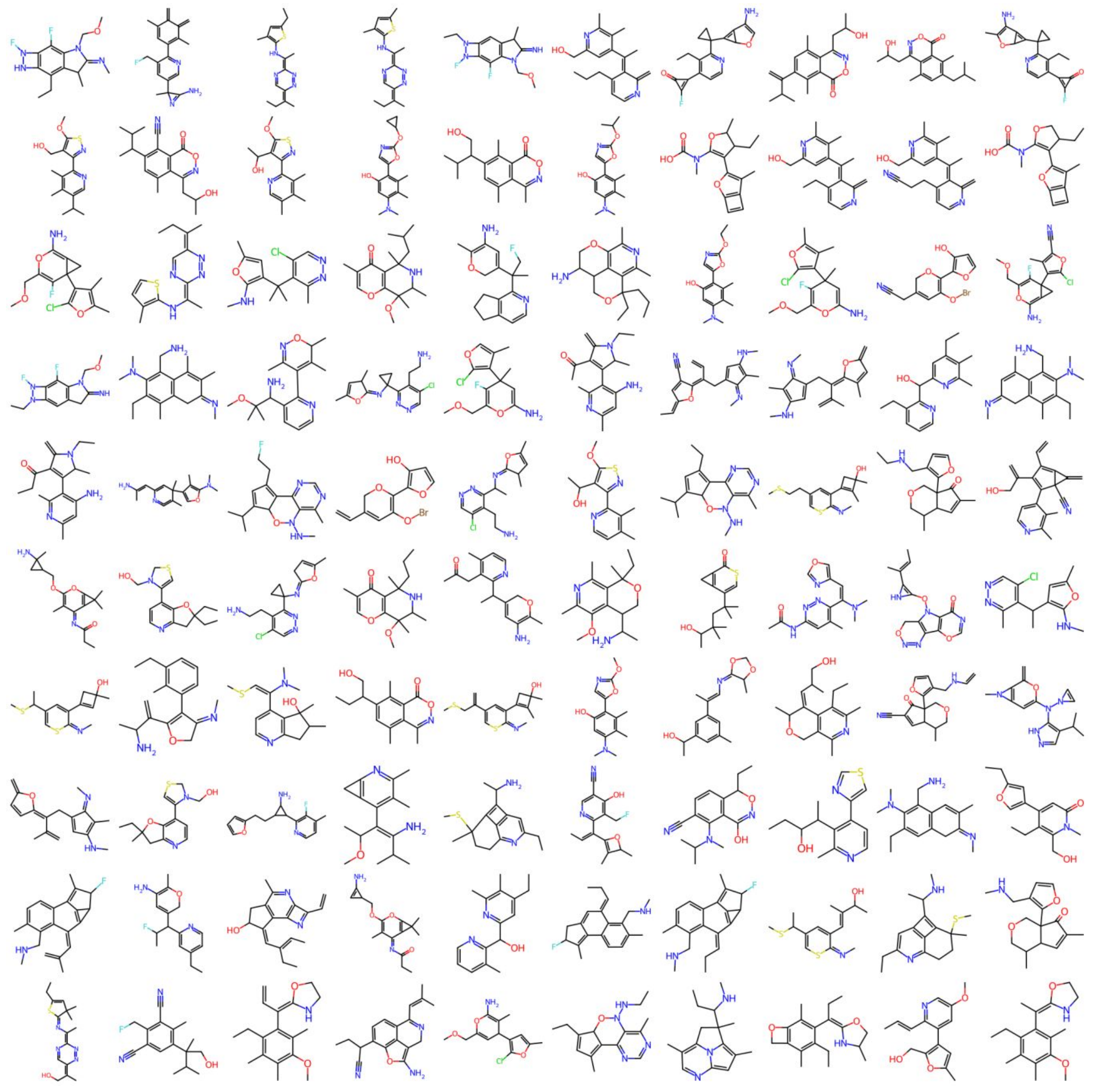

Supplementary Figure 2. A hundred molecules generated by VGAE-MCTS with a QED of 0.9 or higher, starting from those with the highest QED scores.

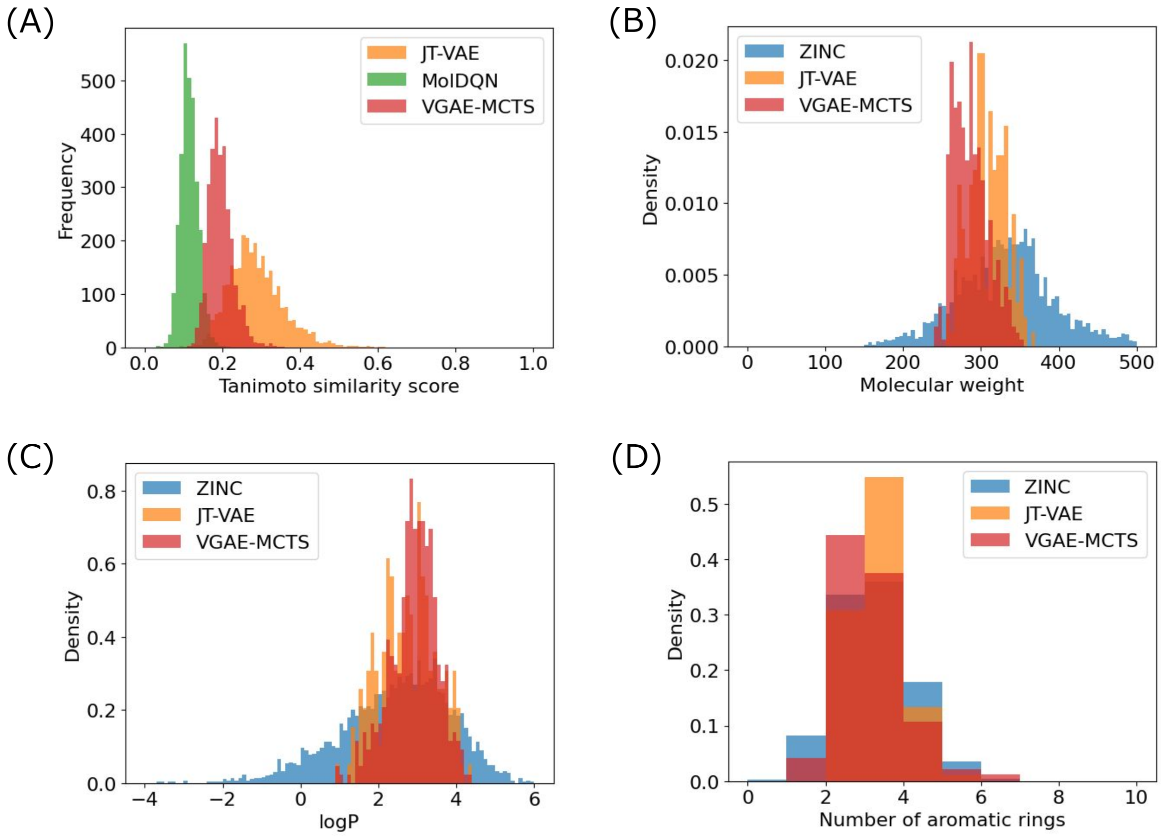

Supplementary Figure 3. The results of structural and physicochemical analyses of molecular generative models. (A) Similarity scores were calculated using the 2,048-dimensional descriptors of ECFP4 with the molecules generated by each method and 3,000 randomly picked ZINC compounds. (B) The results of the molecular weight comparison among the generated molecules optimizing the QED and (3,000 randomly picked) ZINC molecules. (C) The results of the logP value comparison. (D) The results of the number of aromatic rings comparison.
